# Explainable AI-driven intelligent system for precision forecasting in cardiovascular disease

**DOI:** 10.3389/fmed.2025.1596335

**Published:** 2025-07-09

**Authors:** Anas Bilal, Abdulkareem Alzahrani, Khalid Almohammadi, Muhammad Saleem, Muhammad Sajid Farooq, Raheem Sarwar

**Affiliations:** ^1^College of Information Science and Technology, Hainan Normal University, Haikou, China; ^2^Department of Computer Science, Faculty of Computing and Information, Al-Baha University, Al-Baha, Saudi Arabia; ^3^Department of Computer Science, Applied College, University of Tabuk, Tabuk, Saudi Arabia; ^4^Department of Computer Science, Air University, Islamabad, Pakistan; ^5^Department of Cyber Security, NASTP Institute of Information Technology, Lahore, Pakistan; ^6^OTEHM, Manchester Metropolitan University, Manchester, United Kingdom

**Keywords:** cardiovascular diseases, explainable artificial intelligence, electronic medical records, machine learning, shap, lime

## Abstract

**Introduction:**

Cardiovascular diseases (CVDs) are complex and affect a large part of the world’s population; early accurate and timely prediction is also complicated. Typically, predicting CVDs involves using statistical models and other forms of standard machine learning. Although these methods offer some level of prediction, their black-box nature severely hinders the ability of the healthcare professional to trust and use the predictions. The following are some of the challenges that Explainable Artificial Intelligence (XAI) may solve since it can give an understanding of the decision-making system of AI to build confidence and increase usability.

**Methods:**

This research introduced an intelligent forecasting system for cardiovascular events using XAI and addressed the limitations of traditional methods. This proposed system incorporates advanced machine learning algorithms integrated with XAI to examine a dataset comprising 308,737 patient records with features including age, BMI, blood pressure, cholesterol levels, and lifestyle factors. This dataset was sourced from the Kaggle Cardiovascular Disease dataset.

**Results:**

Incorporating XAI offers an understandable explanation so that the healthcare professional can understand and make the AI-driven prediction trustworthy enough to improve the decision-making of treatment and care delivery for the patients. The simulation results of the proposed system provide better results than those of the previously published research works in terms of 91.94% accuracy and 8.06% miss rate.

**Discussion:**

This proposed system makes it clear that XAI has the potential to significantly improve cardiovascular healthcare by enhancing transparency, reliability, and the quality of patient care.

## 1 Introduction

In the past decade heart disease or cardiovascular has remained the leading cause of fatalities in the whole world. The computational prediction of cardiovascular diseases is a critical and complex health issue in reality. It impacts the functionality of blood vessels and causes coronary artery infections that incapacitate the body of the patient, more so the adults and the elderly. According to the World Health Organization (WHO), cardiovascular diseases are the leading cause of death with more than 18 million deaths in the world annually (1). The US spends one billion dollars per day on the treatment of heart diseases (2). These heart diseases (stroke, heart attack, hypertension etc.) are the leading cause of death in America. Consequently, early prognosis of heart disease is very crucial in nursing cardiac patients before they develop a heart attack or a stroke (3).

Cardiovascular diseases can be detected via medical tests and Electronic Medical Records (EMRs) extracted from wearable sensors .Identifying useful risk factors for heart diseases from electronic medical tests is challenging as physicians attempt to give efficiency and accuracy in the diagnosis of the patients (4). These EMRs are unstructured and have been growing in size day by day due to the medical tests on the patients. Wearable sensors are also used to record internal and external body signals as cardiovascular checks for heart disease. However, wearable sensor data that aids in predicting heart diseases contains signal artifacts such as missing values and noise that reduce the system performance and yield inaccurate outcomes (5, 6). To address these challenges, artificial intelligence (AI) has become increasingly central to modern healthcare, offering intelligent systems capable of processing large-scale unstructured data with high precision. Advanced models such as fuzzy deep learning architectures, quantum-enhanced networks, and deep convolutional neural networks have demonstrated success in detecting plant diseases, early-stage cancers, diabetic retinopathy, and periodontal conditions (7–11). These innovations highlight AI’s potential to improve diagnostic accuracy, optimize data interpretation from EMRs and sensors, and support clinical decision-making in cardiovascular care and beyond. Recent studies have demonstrated that exosomal lncRNAs and nanozyme-enhanced amplification techniques offer promising strategies for the diagnosis and treatment of viral myocarditis (12, 13).

Immunomodulatory therapies using engineered extracellular vesicles have also shown potential in targeting multiple pathways involved in cardiac inflammation (14). First of all, integrating wearable sensors and EMRs is a challenging and major approach to managing cardiac patients. Secondly, feature selection of data plays an important role in heart disease prediction, where selecting the most important and useful features from the data is most often difficult. Machine learning models are increasingly being used for cardiac signal analysis and perioperative cognitive disorder prediction, offering improved clinical decision-making tools (15–17). On the molecular level, ALKBH5 and LINC00657 have been identified as key regulators in angiogenesis through post-transcriptional and miRNA-mediated pathways (18, 19). Epidemiological analyses employing large datasets and machine learning approaches have revealed associations between metabolic indicators—such as the triglyceride-glucose index and GPER activity and cardiovascular risks in hypertensive populations (20, 21). As such, these diseases are compounded by their multifactorial nature, hence calling for stringent and timely forecasts to enhance early management. Even today, a science such as clinical cardiology using all the modern possibilities in research and technologies still struggles to predict subsequent cardiovascular events. They include the constantly increasing volume of heterogeneous data, variability of patients’ responses, and complex interactions between genes, behavior, and environment that determine CVD risk (22, 23) .Advances in cardio-oncology have also been highlighted, particularly in the context of panvascular medicine and its therapeutic integration (24). Pediatric research points to a growing concern in early-onset cardiac remodeling and rare syndromic cases treated with innovative device interventions (25, 26). Moreover, systemic inflammatory markers and micronutrient levels, such as neutrophil-lymphocyte ratios and vitamin D, have been linked to cardiovascular mortality and disease prevalence (27, 28).

Nowadays, several systems have been proposed in order to predict and diagnose cardiovascular disease using data mining techniques and hybrid models as reviewed next in the related work section (29–32). These traditional forecasting models in cardiovascular diseases have been developed based on a statistical approach and other classical machine learning techniques (33–35). These methods are mostly based on pattern recognition to make predictions of possible cardiovascular incidents. While they provide a certain level of predictive capability, they tend to operate as “black boxes,” offering little to no insight into how predictions are made (36, 37). This lack of interpretability can cause trust issues with the health care professionals and patients as clinicians need to know why the predictions are made to make an appropriate clinical decision on the patients. Therefore, these conventional models’ inherent opaqueness and non-interpretable nature become a major drawback of their usage in clinical contexts.

Besides, the data used in the conventional models are usually constrained to certain factors while the system requires precise modeling of complicated scenarios. For example, blatant demographic data, and medical history are significant; however, the impact of life conditions, surroundings, and genetic factors cannot be overlooked. The traditional approaches also do not consider the dynamic nature of patient health, where changes in health status and behavior significantly contribute to cardiovascular risk. With this, the traditional models take more of a static position, which can lead to making decisions and providing predictions that are partly accurate and may not be characteristic of the current risks of the patients. Furthermore, many of these models have been developed using small or imbalanced datasets without adequate external validation, raising concerns about their generalizability and robustness across different populations. Such models may overfit the training data and fail to perform well in real-world clinical settings. Additionally, these models often lack mechanisms to incorporate evolving patient data or adapt to changes in patient health over time, limiting their clinical usefulness.

Explainable Artificial Intelligence (XAI) presents the paradigm shift to these challenges. It allows explaining AI decision-making processes to increase their interpretability and transparency (38, 39). This transparency becomes crucial in a clinical context where the potential for AI-based decision-making may either enhance the level of trust or reduce the effectiveness of clinical decisions. By revealing how the AI reaches its conclusions, XAI builds trust and offers deeper insights into the potential and existing factors influencing cardiovascular risk. This aspect of explaining and justifying these predictions can help fill this gap and formally integrate these advanced AI technologies into the clinical environments for better recognition and acceptance.

To enhance the interpretability of the proposed model for cardiovascular disease prediction, this study uses XAI methods like SHAP and LIME. SHAP is derived from cooperative game theory, which provides important values for all features and paints a global and local picture of how each feature affects the model outcomes (40). In contrast, LIME is used as an interpreter to explain individual predictions based on converting a complex model to a simpler one by showing those features whose change influences specific results in a more easily understandable manner (41). These methods improve the credibility and reliability of the discovered patterns to the predictions made by AI, which makes them more understandable to doctors.

The diagnosis of CVDs has been an active area of research interest for several decades. Conventional approaches mainly focus on statistical models and basic or standard pattern analysis methods to establish risk factors and forecast occurrences. Former models still rely on demographic characteristics and medical history, including the Framingham Risk Score. Other established clinical risk scores such as SCORE2 and ASCVD are also commonly used benchmarks in clinical practice. However, these scores often rely on a limited set of variables and assume linear relationships, which may not fully capture the complexity of cardiovascular risk factors. Although useful, these models are often static and lack adaptability to individual patient variability. Furthermore, the complexity of CVDs, influenced by genetic, environmental, and lifestyle factors, requires more advanced methods for accurate predictions. The research work (42) emphasized the importance of machine learning in combating CVDs which are considered significant worldwide challenges. It presented a conceptual AI system that used algorithms such as Support Vector Machine (SVM), K-Nearest Neighbors (KNN), and Random Forest (RF) to improve the efficiency of CVD risk prediction using real-life clinical data. The system’s validity proved its operational employability and efficiency in dispensing risk evaluations. Further, it provided preventive care suggestions concerning specific patients, creating a culture of positive health management. This focused on the predictive value of early CVD diagnosis using machine learning that enabled effective individualized treatment to save patient’s lives and minimize the general costs associated with such diseases. However, the performance of the proposed AI-driven system depends on the quality and coverage of the healthcare dataset used for training and testing, which may be a significant challenge for its wide applicability to diverse populations.

According to Swain (43), the prediction of cardiovascular diseases underlined the importance of using machine learning to enhance healthcare. Logistic Regression (LR), Random Forest, Gradient Boosting, Support Vector Machine (SVM), and Naive Bayes have been commonly explored for integrating cardiovascular health conditions. The research indicated that when Naive Bayes and Decision Trees are used together, their diagnostic accuracy is higher than that of other classifiers. Dealing with noise and missing values is one of the key steps in data pre-processing, and it should be done carefully to improve the model’s performance. Measures such as accuracy, sensitivity, and specificity made it possible to determine whether these models were useful in identifying at-risk individuals. In addition, data visualization techniques were appreciated, and the relationships of numerous attributes that can impact the probability of developing cardiovascular diseases were explained. The literature highlighted the need to embrace intelligent machine learning in combination with sound approaches to handling the data to enhance cardiovascular disease prediction.

In Nagavelli et al. (44), the study focused on the importance of machine learning for predicting CVD, highlighting the application of improved computational tools for better diagnostic capabilities and patient care. They intended to emphasize the significance of altering the baseline and the follow-up online datasets on CVD events. The study also used logistic regression, SVM, naïve Bayes, random forest, and KNN models that were selected depending on their performance in accurately diagnosing the disease. A standard procedure and rigorous validation process were followed, and the reliability of these models was checked to estimate the likelihood of CVD based on data input. The study found that CVD predictions using machine learning techniques reduced classification errors and increased the diagnostic reliability of the information. This multifaceted approach underscored the significant applications of artificial intelligence in clinical decision-making and expanded the current body of knowledge on computational biology and predictive modeling. The research (45), provided a thorough analysis of existing literature on CVD risk prediction models. The review, which included 212 articles from an initial 9965 references, identified 363 multivariable models, predominantly from Europe, with 46% focusing on both fatal and non-fatal coronary heart disease. Most models (58%) predicted risk over 10 years, although 13% lacked a clear prediction horizon. Common predictors were smoking (90%) and age (88%), with 69% of models being sex-specific. Significant methodological heterogeneity was noted, inconsistent definitions of predictors and outcomes were found, and many models were missing critical clinical and methodological details. Validation was limited, with only 36% undergoing external validation and 19% validated by independent investigators, showing varied performance in discrimination and calibration metrics. The authors recommend focusing future research on validating and comparing existing models, adapting them to local contexts, and incorporating new predictors to improve predictive accuracy, highlighting the complexity and need for enhanced methodologies in CVD risk prediction. The research highlighted significant limitations in CVD risk prediction models, including methodological heterogeneity and lack of external validation, undermining their reliability and generalizability in real-world settings.

The study (40) employs SHAP to explain three different ML models used for determining critical clearing time in power systems and to identify the impact of the variables for improved planning and operations. In Wu et al. (41), a deep learning recommender model is used for heart disease and diabetes with LIME explanation. By providing interpretable results involving features CholCheck and HighBP for heart disease, glucose for diabetes, and BMI and age for diabetes, the operation is credible and beneficial for patients.

Table 1 includes several studies that address CVD prediction and diagnosis with the help of different machine-learning techniques and their performances. For instance, Kuhar et al. (42) employed a Random Forest algorithm that yielded 90% accuracy in identifying ASCVD with the optimal utilization of healthcare resources. Nagavelli et al. (44) employed methods such as Logistic Regression and Support Vector Machine and got enhanced diagnostic efficacy. In these works, diagnostic accuracy was improved, but the problem was that XAI did not receive enough attention, and it is essential to improve the trust in these models in the clinical field (47–51).

**TABLE 1:** Comparative analysis of previously published works.

References	Method	Pre-processing layer	Outcome	Decision making
Kuhar et al. (42)	Random Forest (RF)	✓	AUC 0.902 identified 90% of CVD cases by screening 43%	Improved CVD risk prediction and efficient healthcare practices
Nagavelli et al. (44)	LR, SVM, Naive Bayes, RF, KNN	✓	Improved diagnostic accuracy and reduced misclassification	Reliable CVD predictions with rigorous testing and validation
Damen et al. (45)	Various European multivariable models	×	Highlighted heterogeneity and the need for external validation	Recommended model validation and adaptation to local contexts
Nguyen et al. (46)	Genetic Algorithm (GA) + Fuzzy Standard Additive Model (SAM), Genetic SAM (GSAM)	×	Superior medical diagnosis performance at a lower cost	Effective for high-dimensional data, useful in decision support
Latha et al. (47)	Ensemble Classification (Bagging, Boosting)	✓	Increased heart disease prediction accuracy by 7%	Enhanced early-stage disease prediction through improved weak classifiers
Long et al. (48)	Rough Sets + Interval Type 2 Fuzzy Logic System	×	Dominated heart disease diagnosis with fewer features	Supports decision-making in high-dimensional data and uncertainties
Mohan et al. (49)	Hybrid RF with a Linear Model (HRFLM, RF + Decision Tree)	×	Achieved 88.7% accuracy in heart disease prediction	Hybrid models improved cardiovascular disease prediction accuracy
Tuli et al. (50)	Ensemble Deep Learning, Fog Computing, IoT	✓	Efficient real-time heart disease diagnosis in fog environments	Improved healthcare services through secure and reliable fog computing
Samuel et al. (51)	Artificial neural Network (ANN) + Fuzzy Analytic Hierarchy Process (AHP)	✓	Achieved 91.10% accuracy, outperforming conventional methods	Enhanced HF risk prediction with attribute weight consideration

This research addresses the critical limitations of current computational models for predicting cardiovascular diseases, particularly their “black box” nature and lack of explainable features, which hinder their widespread adoption in clinical practice. Based on the state of the art presented, there is a clear need for a study that bridges the gap between AI-driven predictions and their reliable application in healthcare. By integrating Explainable AI (XAI) techniques such as SHAP and LIME, this study aims to develop an intelligent forecasting system that improves prediction accuracy and offers transparent and interpretable insights. These explainable features will empower clinicians to make more informed decisions, enhancing the trustworthiness and utility of AI predictions in real-life healthcare settings

## 2 Materials and methods

Cardiovascular disease risk assessment and healthcare management have made significant strides toward a sophisticated predictive capability; however, this field is plagued by several major issues that hinder the development and application of accurate prediction models. One major challenge is the complexity of CVD, which includes one or several risk factors and comorbidity factors that differ in different population groups. This complexity makes it difficult to develop models that balance realism with specificity. Additionally, the variability and quality of patient data present another hurdle, as data often come from diverse sources with different levels of accuracy and completeness, leading to potential biases and inconsistencies in model predictions. Furthermore, implementing artificial intelligence into clinical practice can threaten the interpretability of AI decision-making. Clinicians must be confident in the outputs produced by these models, especially when it comes to cardiovascular risk assessments. The absence of proper explainability architectures in many AI models is another problem that hampers their adoption and implementation in various contexts.

To address these challenges, explainable AI (XAI) must be integrated, which can act as a solution. XAI helps make the AI models better understandable by providing ways to interpret how the AI model predicts. It lifts clinicians’ comfort because they are able to know why a model has made a certain decision while preventing the use of unsuitable models in practice. In this research work, a model is proposed to make AI models more transparent and understandable, where XAI facilitates their integration into healthcare settings, enabling the development of intelligent forecasting systems that are not only accurate but also reliable, ethical, and widely accepted.

### 2.1 Proposed model components

**FIGURE 1:**
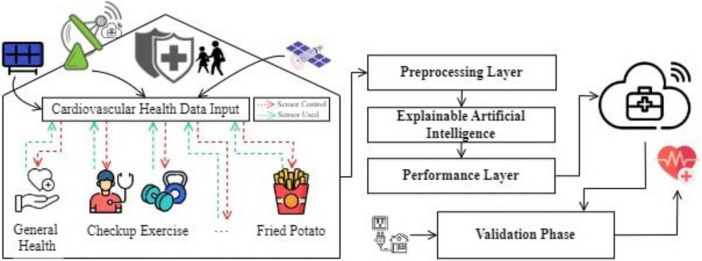
Abstraction of the proposed model.

Figure 1 illustrates the abstraction of the proposed model for CVD prediction, which consists of five key components: input layer, pre-processing layer, training layer integrated with XAI, performance layer, and validation step.

• *The input layer:* Enables devices used in healthcare to become more efficient by communicating sensory data (wirelessly), potentially negating cellular communication costs for those same products. The service-oriented layer provides data collection and transmission, crucial for full-scale healthcare management.
• *Pre-processing layer:* This layer is employed to manipulate the data from input layers with the help of averaging smoothing, normalization and cleaning techniques to make it more factual before modeling. Processed data is saved on the cloud for future reference.
• *Training layer with XAI:* Train data is again split into the 80% for training and 20% of testing. This is data used to train machine learning models on it. It combines XAI methods like SHAP & LIME to generate human-friendly explanations about the model predictions and make them transparent andd interpretable.
• *Performance layer:* After training, evaluate the model performance and store results in cloud-based data storage.
• *Validation step:* The model will get the learned data from cloud storage to predict how heart disease will progress now. It is a constantly learning and evolving system that learns from previous predictions to make better decisions over time.

### 2.2 Prediction model using XAI

**FIGURE 2:**
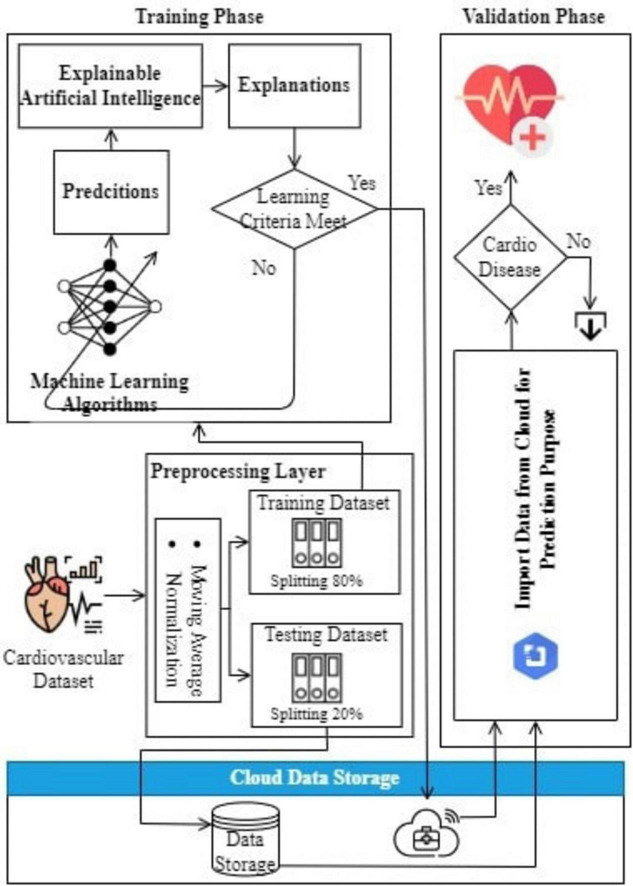
Proposed model.

Figure 2 shows the model to predict cardiovascular disease using XAI. Data from various sensors and healthcare devices in a dataset (52) are acquired by this model and passed through the pre-processing layer for averaging smoothing, normalization, and data cleaning to make the data accurate and relevant. Missing numerical values were handled using mean imputation, and categorical features were imputed using mode imputation where needed. Feature selection was based on feature importance scores from tree-based models. The pre-processed data is divided into two subsets: Training Data Set: 80% and Testing Data Set: 20%. The training data is then used to develop predictive models based on several machine learning methodologies; on the other hand, the testing dataset is kept safely in the cloud storage.

#### 2.2.1 Explain ability using SHAP and LIME

The predictions generated by the machine learning models are then subjected to XAI methods, which provide human-friendly explanations, enhancing transparency and interpretability of the outcomes. The primary goal of XAI in this context is to build trust using SHAP and LIME in machine learning models (often considered “black boxes”) by highlighting the importance of local and global variables through post hoc explanations. XAI not only helps clarify the black-box nature of these models but also advocates for accountable AI by promoting the development of transparent models. Making the decision process of Machine Learning models more interpretable to the end-users and other stakeholders is a central goal of explainable AI.

LIME and SHAP are the model-agnostic interpretation approaches that keep high prediction performance and the distinction between the model and the explanation. LIME offers local explanations that explain the model’s behavior in the proximity of a certain example, while SHAP provides global explanations showing the impact of features in general on the predictions. These methods enhance the contribution of local and global variables in the decision-making process of the machine learning models to be in a format that users and other stakeholders will easily understand.

*LIME:* It is a well-known approach for local explanation that builds local surrogate models that can explain complex machine learning models. It does so by initially reprocessing the data into a new dataset, using this newly generated data set to train the interpretable model. To attain this aim, the loss function *L* is used to minimize the optimality between the predictions of the original model *f* as well as the interpretable surrogate model *g*. The function is expressed as:


(1) $$\gamma \,\left(x\right)=a\,r\,g_{g\,\epsilon \,G}^{m\,i\,n}\,\left(L\,\left(f,g,\pi_{x}\right)+\Omega \,\left(g\right)\right)$$

In Equation 1, the loss function *L(f,g*π_*x*_) involves the original model *f*, the interpretable surrogate model *g*, and π_*x*_, which represents the input features corresponding to instance *x*. This function aims to minimize the difference between the predictions of the two models while incorporating a regularization term Ω(*g*). This local explanation model can also be used to explain individual predictions, which could help explain why this specific decision was made in this instance.

*SHAP:* SHAP is a global explanation technique that helps the user quantify any feature’s contribution to the formation of the model’s decision. This is based on the theory of Shapley values used in cooperative game theory, here the worth of every feature is computed for all possible scenarios of feature values. The formula for calculating the Shapley value ϕ_*j*_(*x*) is as follows:


(2) $$\begin{array}{c} \varphi_{j}(x)=\sum_{s}\subseteq \{x_{1},x_{2},...,x_{m}\}\backslash \{x_{j}\}\frac{|s|!(m-|s|-1)!}{m!}\\ \left(val(s\cup \{x_{j}\}-val(s)\right) \end{array}$$

In Equation 2, the Shapley value φ_*j*_(*x*) Incorporates parameters such as *s*, which represents subsets of features excluding *x*_*j*_, and *m*, the total number of features. The term *val*(*s* ∪ {*x*_*j*_} − *val*(*s*) quantifies the contribution of feature *x*_*j*_ to the prediction for the subset *s*. SHAP values can be calculated to give an overall global explanation of how each feature impacts the prediction and other scalable models as well.

*Partial dependence plots*: It exhibits a single feature and how it affects predictions, as PDP does. The partial dependence function ${\hat{f}}_{\left(x_{s}\right)}$ is defined as:


(3) $$\hat{f}(x_{s})\frac{1}{n}\sum {}_{i=1}^{n}f(x_{2},x_{i}^{c})$$

In Equation 3, the partial dependence function ${\hat{f}}_{\left(x_{s}\right)}$ is defined over *n* instances of the dataset, where *x*_*s*_ represents the specific feature of interest and $x_{i}^{c}$ Denotes the complementary features. This function computes the average prediction by fixing the value of *x*_*s*_ while varying $x_{i}^{c}$. It offers a graphical view that makes it possible to comprehend the global relationship between the features and the outcomes predicted by PDP.

Subsequently, the predetermined criteria are checked in order to ascertain if all the learning objectives have been met after which the trained explanation patterns are tested for validity. If the criteria are met, the trained model is saved on the cloud; otherwise, the machine learning algorithm is recursively retrained until the criteria are met. In the validation phase, the testing dataset and the learned explanation patterns are imported from the cloud to predict cardiovascular disease. These predictions are then re-evaluated, and if the model is able to detect possible cardiovascular risks, the system will send out an alert signifying the existence of the disease. If no risks are identified, the process is discarded.

## 3 Results

Integrating XAI into intelligent forecasting systems presents a transformative approach in the rapidly evolving field of cardiovascular disease prediction. This research aims to model an intelligent forecasting system that predicts cardiovascular risks with better performance and provides clear, interpretable insights into the decision-making process. By leveraging advanced machine learning techniques combined with XAI, this proposed system enhances the transparency and trustworthiness of predictions, enabling healthcare professionals to understand better the underlying factors driving each prediction. This approach aims to bridge the gap between complex AI models and their clinical applicability by applying XAI on a dataset, where 80% is used for training and 20% for testing. This ensures that predictions are accurate and explainable, making them reliable for critical healthcare decisions.

**FIGURE 3:**
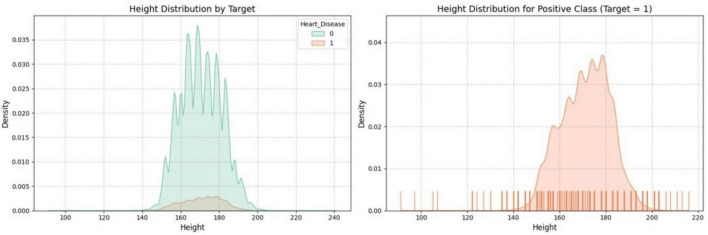
Distribution of height by target variable.

Figure 3 provides a distribution of the “Height” feature concerning a target variable through two key plots. The left plot shows the density distribution of “Height” for both target variable classes, using distinct colors to facilitate easy comparison between the two groups. The second plot on the right focuses exclusively on the positive class (where the target = 1), offering a more granular view of its distribution. This plot includes a filled density curve and a rug plot along the x-axis, highlighting individual data points, giving insight into the concentration and spread of “Height” within the positive class.

**FIGURE 4:**
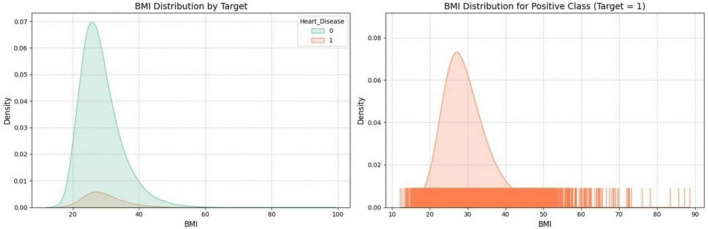
Distribution of weight by target variable.

Figure 4 visualizes the distribution of the “Weight” feature through two key plots. One plot shows the density distribution of “Weight” for both classes of a binary target variable, while the other focuses specifically on the distribution of “Weight” within one class, like the positive class. The plots use distinct colors to differentiate between classes, which helps understand the relationship between “Weight” and the target variable within the dataset.

**FIGURE 5:**
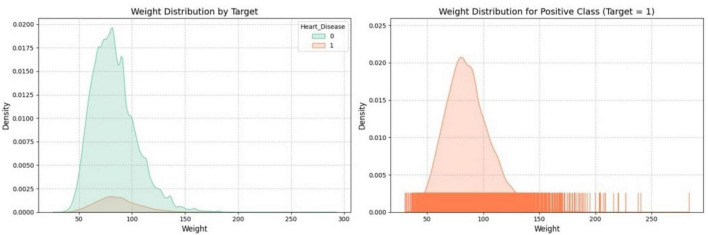
Distribution of BMI by target variable.

Figure 5 presents the distribution of Body Mass Index (BMI) in relation to a binary target variable, likely representing the presence or absence of heart disease. The left plot compares the BMI distribution for both target classes, with distinct colors highlighting the differences between those with and without heart disease. The right plot zooms in on the BMI distribution specifically for the positive class (where the target variable = 1, possibly indicating heart disease), offering a more detailed view with a filled density curve and a rug plot showing individual data points.

**FIGURE 6:**
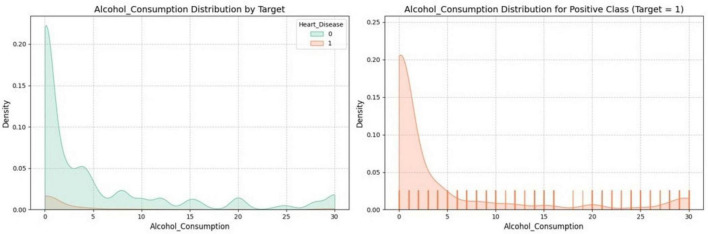
Distribution of alcohol-consumption by target variable.

Figure 6 shows the distribution of alcohol consumption in relation to a binary target variable, likely indicating the presence or absence of heart disease. The left plot compares the alcohol consumption distribution between both target classes, using different colors to distinguish between those with and without heart disease. The right plot focuses solely on the positive class (where the target variable = 1, possibly indicating heart disease), providing a detailed view of alcohol consumption within this group with a density curve and individual data points highlighted by a rug plot.

**FIGURE 7:**
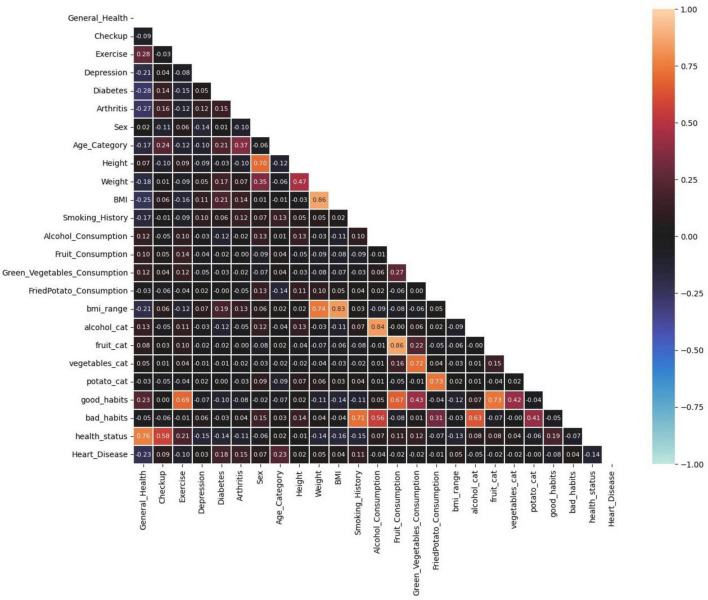
Pearson correlation.

Figure 7 shows a Pearson correlation matrix, illustrating the linear relationships between different features in the dataset. Each cell represents the correlation coefficient between two variables, with values ranging from −1 (strong negative correlation) to 1 (strong positive correlation). The color gradient highlights the strength and direction of these correlations, with warmer colors indicating stronger correlations and cooler colors indicating weaker ones. This matrix helps quickly identify significant relationships between variables.

Table 2 provides the confusion matrix for six machine learning models (Decision Tree, Random Forest, Multi-Layer Perceptron, XGBoost, LightGBM, and Catboost) across training (246,989 samples) and testing (61,748 samples) datasets. The True Positive (TP) counts are consistently high, ranging from 226,307 to 226,679 for training and 56,532 to 56,667 for testing, indicating strong performance in correctly identifying positive cases. However, the True Negative (TN) counts are significantly lower, with values ranging from 514 to 1,028 for training and only 103 to 191 for testing, suggesting that the models are less effective at identifying negative cases. The False Positive (FP) counts range from 18,942 to 19,548 for training and 4,802 to 4,890 for testing, while the False Negative (FN) counts are relatively low, ranging from 340 to 712 for training and 88 to 223 for testing. These facts highlight the models’ strengths in predicting positives and reveal challenges in accurately identifying negatives.

**TABLE 2:** Confusion matrix and the performance matrices of the proposed system.

**Confusion Matrix**												
	Decision Tree		Random Forest		Multi-Layer Perceptron		XGBoost		LightGBM		Catboost	
	Train (246989)	Test (61748)	Train (246989)	Test (61748)	Train (246989)	Test (61748)	Train (246989)	Test (61748)	Train (246989)	Test (61748)	Train (246989)	Test (61748)
**TP**	226443	56555	226307	56532	226563	56637	226642	56651	226679	56667	226631	56640
**TN**	892	171	1028	191	514	130	522	113	422	103	606	118
**FP**	19078	4822	18942	4802	19456	4863	19448	4880	19548	4890	19364	4875
**FN**	576	200	712	223	456	118	377	104	340	88	388	115
**Performance Matrices**												
**ACC**	92.04	91.87	92.04	91.86	91.94	91.93	91.97	91.93	91.95	91.94	92.00	91.92
**TPR**	99.75	99.65	99.69	99.61	99.80	99.79	99.83	99.82	99.85	99.84	99.83	99.80
**TNR**	4.47	3.42	5.15	3.83	2.57	2.60	2.61	2.26	2.11	2.06	3.03	2.36
**FNR**	7.96	8.13	7.96	8.14	8.06	8.07	8.03	8.07	8.05	8.06	8	8.08
**FPR**	95.53	96.58	94.85	96.17	97.43	97.40	97.39	97.74	97.89	97.94	96.97	97.64
**LR+**	1.044	1.031	1.051	1.035	1.024	1.024	1.025	1.021	1.020	1.019	1.029	1.022
**LR-**	1.780	2.377	1.545	2.125	3.136	3.103	3.076	3.570	3.815	3.912	2.640	3.423
**PPV**	92.23	92.14	92.28	92.17	92.09	92.09	92.10	92.07	92.06	92.06	92.13	92.08
**NPV**	60.76	46.09	59.08	46.14	52.99	52.42	58.06	52.07	55.38	53.93	60.97	50.64

**FIGURE 8:**
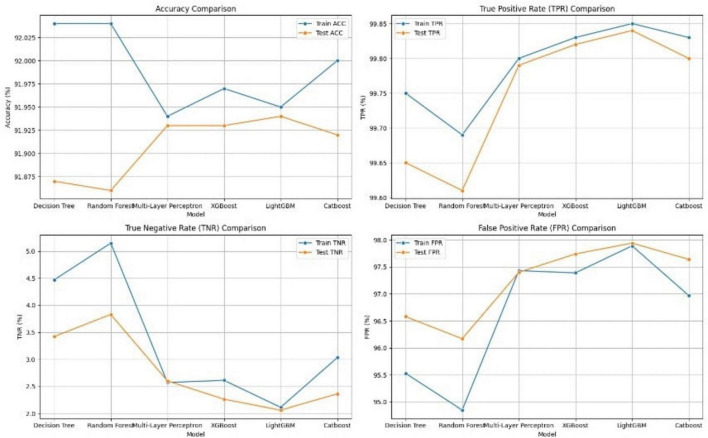
Performance matrices of the proposed system.

Table 2 and Figure 8 also compare the performance of these machine learning models across several key metrics, including Accuracy (ACC), True Positive Rate (TPR), True Negative Rate (TNR), False Negative Rate (FNR), False Positive Rate (FPR) predictive values (PPV and NPV), Likelihood Positive Ratio (LR+), and Likelihood Negative Ratio (LR-). All models show high accuracy and TPR, indicating strong performance in identifying positive cases. Among all models, LightGBM achieves the highest accuracy on the testing set at 91.94%, making it the recommended algorithm for this task.

**FIGURE 9:**
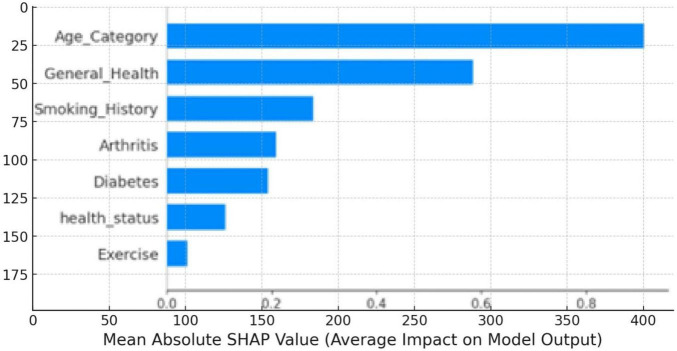
SHAP summary plot value impact on model output magnitude.

**FIGURE 10:**
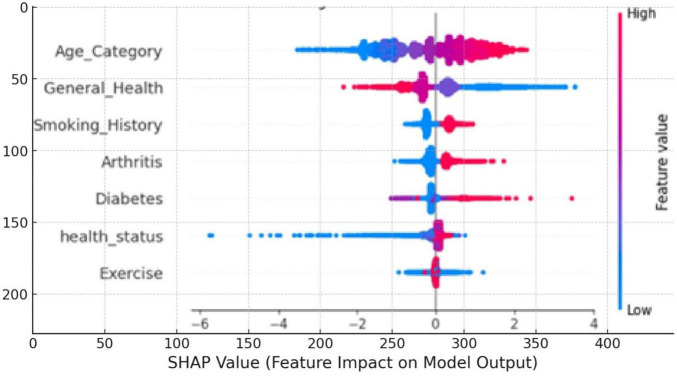
SHAP beeswarm plot value impact on model output.

**FIGURE 11:**
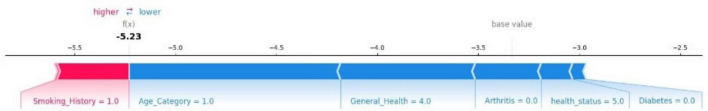
Feature contributions to cardiovascular disease risk prediction: a SHAP waterfall analysis.

**FIGURE 12:**
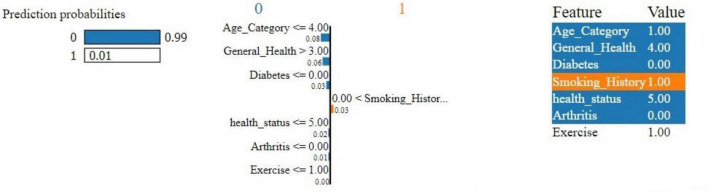
LIME explanation of the proposed system.

The SHAP and LIME plots in Figures 9–12 were generated for the LightGBM model to visualize the impact of each feature on cardiovascular disease prediction. Figure 9 is a SHAP summary plot for the testing set, showing the average impact of each feature on the model’s output. The length of each bar represents the mean absolute SHAP value, indicating the importance of each feature. “Age_Category” has the highest impact on the model’s predictions, followed by “General_Health” and “Smoking_History.” This plot helps to understand the most influential features in the model’s decision-making process.

Figure 10 is the SHAP beeswarm plot illustrating the impact of various features on the model’s predictions for cardiovascular disease. Age_Category, General_Health, and Smoking_History are the most influential factors, with higher values (shown in red) generally increasing the likelihood of a positive prediction for cardiovascular disease. The plot highlights how individual features contribute to the model’s output, with age being the most significant predictor.

Figure 11 illustrates the SHAP waterfall plot, showing the feature contributions to a specific individual’s cardiovascular disease risk prediction. The true outcome—absence of cardiovascular disease—is included to provide context for the model’s prediction. The model predicts a final score of −5.23, indicating a low risk of cardiovascular disease for this individual. Smoking_History = 1.0 increases the risk, pushing the score higher, while Age_Category = 1.0, General_Health = 4.0, and the absence of Arthritis and Diabetes lower the risk, pushing the score further down. This clearly shows how each feature contributes to the final prediction score, moving it toward a lower-risk outcome.

Figure 12 is a Local Interpretable Model-Agnostic Explanations (LIME) explanation showing different features contributing to the model’s prediction for cardiovascular disease risk. The figure reveals that the model predicts a 99% probability that the individual does not have the disease (class 0) and only a 1% probability that they do (class 1). Key features such as Age_Category = 1.00, General_Health = 4.00, the absence of Diabetes and Arthritis, and a good health_status = 5.00 strongly influence the prediction toward class 0, significantly lowering the perceived risk. Although Smoking_History = 1.00 slightly increases the likelihood of class 1, its impact is not enough to outweigh the other features. This LIME explanation effectively demonstrates how each feature influences the model’s prediction, making the decision-making process transparent.<H1>: 4 Discussion

The performance of machine learning models in identifying cardiovascular diseases (CVDs) is crucial for their practical utility. Previous models have shown potential, but their non-interpretability can often nullify any benefit as they are too hard to implement. This research gap is addressed by incorporating Explainable AI (XAI) in the proposed system, which connects advanced machine learning practices with their hands-on usage within healthcare. The proposed model achieves accurate predictions and produces disentangled, explanatory factors influencing decision-making. This transparency builds trust between healthcare professionals and AI systems by clarifying the rationale behind each prediction. The system is designed to integrate with Electronic Medical Records (EMRs) and dashboards, enabling clinicians to access real-time predictions during both routine visits and emergencies. Furthermore, the interpretable outputs help clinicians focus on modifiable risk factors like smoking and BMI, enabling personalized prevention strategies and potentially reducing unnecessary diagnostic tests, thereby enhancing patient care quality.

Although the dataset used in this study (52) includes features like height, weight, and lifestyle factors, it does not provide detailed demographic or genetic profiles. We acknowledge this limitation and applied standard pre-processing techniques, including stratified sampling and cross-validation, to balance representation. Future work will focus on incorporating more diverse datasets to enhance model generalizability and address potential biases. We also acknowledge that model performance may vary across different healthcare systems and populations with varying access to technology.

As shown in Table 3, the proposed XAI-based system outperforms other models. Although previous methods, i.e., Ensemble Classification (47), Hybrid Models (49), and Artificial Neural Networks (ANN), have accuracy rates between 85 and 91%, the proposed system was able to perform at an overall rate of pp more accurately than that with rate of The following section presents a detailed discussion on model performance compared not only with baseline random level but also associated works. This shows how well the model works. While their Likelihood Ratios (LRs) for true positives and negatives are quite similar to the other models, using XAI techniques gives a significant advantage as it makes it transparent whether these animals can be sacrificed or should never be discharged.

**TABLE 3:** Comparison of the proposed system performance with previously published approaches.

References	Model	Accuracy (%)	Miss-rate (%)
Nguyen et al. (23)	GA + Fuzzy SAM (GSAM)	78.7	21.3
Latha et al. (47)	Ensemble Classification (Bagging, Boosting)	85.4	14.6
Long et al. (48)	Rough Sets + Interval Type 2 Fuzzy Logic System	86	14
Mohan et al. (49)	Hybrid Models (HRFLM, RF + Decision Tree)	88.4	11.6
Tuli. et al. (50)	Ensemble Deep Learning, Fog Computing, IoT	89	11
Samuel et al. (51)	ANN + Fuzzy AHP	91	9
Otoom et al. (55)	Naïve Bayes	84.5	15.5
	SVM	84.5	15.5
	Functional trees	84.5	15.5
Vembandasamy et al. (56)	Naïve Bayes	86.4	13.6
Chaurasia e al. (57)	J48	84.35	15.65
	Bagging	85.03	14.97
Parthiban et al. (58)	Naïve Bayes	74	26
Dwivedi et al. (59)	Naïve Bayes	83	17
	Classification tree	77	23
	K-NN	80	20
	Logistic regression	85	15
	SVM	82	18
	ANN	84	16
Mienye et al. (53)	Randomized decision tree ensemble	93	7
Dritsas et al. (54)	Synthetic minority oversampling technique (SMOTE)	87.7	12.3
Proposed XAI model		91.94	8.06

By using SHAP and LIME together, we can see how the model decides on much deeper layers rather than just pointing to its accuracy. Even if the LRs result in minor improvements, they more than make up that difference; however, explaining what each feature represents for classification is excellent. We can determine that the SHAP summary plot and beeswarm plot demonstrate in plain sight the importance of features such as Age_Category, General_Health, or Smoking_History on the model predictions. This will allow healthcare providers to identify and focus on the factors with real impact, significantly improving the model’s utility for making critical clinical decisions.

Furthermore, these explanations are brought to life with local insights through LIME visualization that apply on individual predictions. In this case, Figure 12 demonstrates how LIME opens the “black box” regarding model behavior on example people to explain its rationale for these predictions. This level of interpretability is significant for clinical use, where the reasoning behind a prediction can guide decision-making and ultimately impact individual care.

Table 3 shows that the model not only performed well but could also be a strong tool for clinical use when coupled with its interpretability. Although the Randomized Decision Tree Ensemble (53) and SMOTE-based approaches (54) are within this accuracy range, they lack an explanation for decision-making besides the input value sent in the index cases. What makes the proposed model different from traditional black-box methods is its high predictive power while being simultaneously humanly interpretable. Additionally, while the Framingham Risk Score and echocardiography remain valuable clinical tools, the proposed XAI system can complement these by providing transparent, patient-specific risk explanations that integrate with existing risk assessments. This synergy empowers clinicians to combine AI-driven insights with established clinical protocols for more informed, personalized care.

The proposed system is an essential leap in CVD prediction due to the real-time nature of predictions and explanations for these predictions. This gives the model an accuracy of 91.94%. Because it uses XAI techniques, besides being very effective in identifying patients at risk, any clinician can trust that this is occurring assertTrue. The addition of interpretability through SHAP and LIME enables healthcare providers to visualize the factors driving each decision, turning your model into a working instrument for daily clinical practice. The model may be further refined by improving data inputs (Lifestyle Risk factors) or generalizability of the input using a larger dataset. Still, this method predicts CARDIoGRAMplusC4D future CVD risk better than all standard published models across diverse populations.

The proposed model competes with and even outperforms. Table 3 shows that the proposed system is a competitive achievement and has a greater interpretability ability than many existing models. With the integration of XAI, this system becomes an explainable and trustworthy high-performing healthcare tool for real-world applications with imperviousness to transparency & reliability. Its capability to provide transparency and prescriptiveness for the prediction process distinguishes it as a significant advancement in CVD prediction.

## 5 Conclusion

The major issues in cardiovascular disease detection include difficulties in model interpretability, model applicability across different populations, and high accuracy requirements. These factors hamper the adoption of proper healthcare intervention strategies; hence, to solve these problems, it is necessary to contribute to early diagnosis, lower mortality rates, and universal availability of healthcare services worldwide. To overcome these challenges, this study proposes an XAI-based model that is highly accurate but also explainable and interpretable, allowing healthcare workers to understand why the model makes specific predictions. With the implementation of XAI, the proposed system is designed to perform effectively across different populations, ensuring its applicability on a global scale. By addressing the limitations of previous models, this system improves cardiovascular disease detection and makes cutting-edge healthcare technologies more accessible and dependable worldwide. The proposed system achieves better results than previously published approaches, regarding 91.94% accuracy and 8.06% miss rate.

## Data Availability

The dataset used in this study is publicly available and can be accessed at: https://www.kaggle.com/code/sid4ds/cardiovascular-disease-risk-prediction/input.
